# Heterosynaptic Regulation of α2A-Adrenoceptors on Glutamate/GABA Release in the Prefrontal Cortex of Rats

**DOI:** 10.3390/biomedicines13061322

**Published:** 2025-05-28

**Authors:** Yaru Wei, Yuhan Jiao, Xiaoting He, Xiaodong Tao, Baoming Li, Xuehan Zhang

**Affiliations:** 1 State Key Laboratory of Medical Neurobiology, MOE Frontiers Center for Brain Science, Institutes of Brain Science, Fudan University, Shanghai 200032, China; 2 Department of Physiology and Institute of Brain Science, School of Basic Medical Sciences, Hangzhou Normal University, Hangzhou 310023, China; bmli@hznu.edu.cn

**Keywords:** NE, guanfacine, mEPSC, mIPSC, synaptic transmission, medial prefrontal cortex, rat

## Abstract

**Background/Objectives:** Norepinephrine (NE) plays a crucial role in modulating cognitive processes via α2A-adrenoceptors (α2A-ARs) within the prefrontal cortex (PFC), an essential brain region responsible for higher cognitive functions. The α2A-ARs are found on both postsynaptic and presynaptic membranes in the PFC. Previous studies have shown that presynaptic α2A-ARs, predominantly located at NE terminals, function as autoreceptors that inhibit NE release. However, the expression of α2A-ARs at non-NE terminals, such as glutamate and GABA, remains ambiguous. To clarify the expression patterns and potential roles of α2A-ARs at non-NE terminals, we investigated their presence at the axon terminals of excitatory glutamate neurons and inhibitory GABA neurons in the rat PFC using immunofluorescence double-labeling, whole-cell patch-clamp recordings, and pharmacological approaches. **Methods**: To clarify the expression patterns and potential roles of α2A-ARs at non-NE terminals, we investigated their presence at the axon terminals of glutamate neurons and GABA neurons in the rat PFC using immunofluorescence double-labeling, whole-cell patch-clamp recordings, and pharmacological approaches. **Results**: Our findings delineated the distribution of α2A-ARs at the axon terminals of both glutamate and GABA neurons, and the expression of α2A-AR in the pyramidal neurons within the rat PFC as well. Furthermore, we employed the selective α2A-AR agonist guanfacine to assess the functional role of presynaptic α2A-ARs at these non-NE terminals. Following the application of the PKA inhibitor PKI_5–24_ to block postsynaptic α2A-AR function, guanfacine still significantly decreased the frequency (not the amplitude) of miniature excitatory postsynaptic currents (mEPSCs) and miniature inhibitory postsynaptic currents (mIPSCs) in layer 5–6 pyramidal neurons. Notably, the frequency reduction induced by guanfacine persisted even after the depletion of presynaptic NE vesicles. **Conclusions**: These findings offer a comprehensive analysis of presynaptic α2A-AR expression and function in the PFC, revealing for the first time their role as heteroreceptors that modulate the release of glutamate and GABA. Our results provide morphological and electrophysiological insights into a potential mechanism through which α2A-AR stimulation enhances cognitive functions.

## 1. Introduction

The prefrontal cortex (PFC) is essential for higher cognitive functions, including attention regulation, behavioral inhibition, learning, and working memory [1,2,3,4,5]. Abnormalities in this region are implicated in various psychiatric disorders, such as poor working memory, distractibility, impulsivity, hyperactivity, depression, and anxiety [6,7,8,9,10]. Among the myriad neurotransmitter systems that influence PFC function, norepinephrine (NE) is a key mediator of PFC cognitive functions [11,12,13].

It has been well-established that optimal levels of NE can positively influence prefrontal functions by activating α2-adrenoceptors (α2-ARs) [14,15,16,17]. Activating α2-ARs, particularly α2A-adrenoceptors (α2A-ARs) in the PFC, improved working memory performance [13,18]. Conversely, blocking PFC α2A-ARs decreased working memory performance and increased hyperactivity [19,20]. Additionally, the knockout of α2A-ARs significantly hinders working memory capabilities and negates the cognitive enhancement effects of guanfacine, a selective agonist of α2A-ARs [21].

Previous research has indicated that exogenous norepinephrine (NE) and α2A-AR agonists positively impact PFC functions by acting on postsynaptic α2A-ARs. Notably, the stimulation of α2A-ARs continues to enhance cognitive functions, even in cases where presynaptic NE vesicle components are destroyed or depleted [14,22,23]. Immunoelectron microscopy studies have confirmed that α2A-ARs are predominantly located on dendritic spines and are specifically distributed on the postsynaptic membrane within the PFC [24]. Stimulation of α2A-ARs strengthens working memory by inhibiting cyclic adenosine monophosphate (cAMP) levels, closing hyperpolarization-activated cyclic nucleotide-gated (HCN) channels, and improving the functional connectivity of PFC networks [12]. Additionally, electrophysiological and two-photon imaging studies have shown that the α2A-AR agonist guanfacine decreases hyperpolarization-activated currents (Ih) in the dendrites of pyramidal neurons, which increases the possibility of dendritic Ca^2+^ events in the tuft region and lowers the threshold for dendritic Ca^2+^ spikes, thereby strengthening the functional connectivity of PFC networks [25,26].

In addition to their postsynaptic roles, numerous studies have demonstrated that α2-ARs are located on noradrenergic neurons as presynaptic autoreceptors that inhibit the exocytosis of NE, which provides a negative feedback mechanism that regulates NE release [27]. Within the PFC, the presynaptic α2A-ARs are predominantly recognized for the autoreceptor function [28]. However, it is essential to know that α2-ARs, particularly α2A-ARs, are also expressed as heteroreceptors on non-noradrenergic neurons in the nervous system. They can inhibit the release of many neurotransmitters, including serotonin [29,30,31], dopamine [32], glutamate [33,34,35], and gamma-aminobutyric acid (GABA) [36,37].

Previous electrophysiological studies have demonstrated that α2A-ARs regulate glutamate transmission in the rat PFC and other brain regions through postsynaptic receptor activation and presynaptic mechanisms at non-NE glutamate terminals [34,35,38,39,40]. Also, emerging evidence suggests that presynaptic α2-ARs at non-NE GABA terminals regulate GABAergic transmission in the cerebellar cortex and brainstem [40,41]. Also, immunofluorescence double-labeling revealed the abundant expression of α2A-ARs in the GABAergic interneurons of the rat PFC [42]. Moreover, α2A-ARs in the rostral ventrolateral medulla (RVL) and hippocampus were found on axons and axon terminals that lacked tyrosine hydroxylase (TH) immunoreactivity, a marker for both dopaminergic and noradrenergic terminals [43,44], α2A-ARs in the bed nucleus of the stria terminalis (BNST) co-labeled with the glutamatergic terminal marker [35], indicating the expression of α2A-ARs on non-NE axon terminals. However, in the PFC, the specific expression patterns of α2A-ARs at non-NE glutamate and GABA terminals and their functional involvement in neurotransmitter release regulation remain to be fully elucidated.

To bridge this knowledge gap, we systematically investigated the localization and functional properties of α2A-ARs expressed on the axonal terminals of non-NE neurons, such as glutamate and GABA neurons in the rat PFC. We detected α2A-AR immunoreactivity specifically localized to these presynaptic terminals through dual immunofluorescence labeling. Electrophysiological characterization via whole-cell recordings demonstrated that selective activation of presynaptic α2A-ARs significantly suppressed miniature excitatory (mEPSCs) and inhibitory (mIPSCs) postsynaptic current frequencies. These modulatory effects persisted under presynaptic NE vesicle depletion conditions, confirming receptor functionality independent of endogenous NE release, indicating a heterosynaptic regulation of α2A-ARs on glutamate and GABA release in the PFC.

Our findings comprehensively examine α2A-AR expression in the PFC and uncover their functions as heteroreceptors that regulate the release of glutamate and GABA.

## 2. Materials and Methods

Male Sprague–Dawley rats (30 days old) were purchased from SLACCAS (Shanghai, China) and were housed in a 12/12 light/dark cycle with food and water ad libitum. All the experimental protocols followed the guidelines published in the NIH Guide for the Care and Use of Laboratory Animals (1996) and were approved by the Ethical Committee of Animal Experiments at the School of Basic Medical Sciences, Fudan University (Shanghai, China). We tried to minimize the number of animals used and their suffering.

### 2.1. Chemicals

All reagents were purchased from the Sigma Chemical Company (St. Louis, MO, USA) with the exceptions of guanfacine hydrochloride from Wyeth-Ayerst Company Ltd. (Princeton, NJ, USA) and PKI_5–24_ from the Tocris company (Ellisville, MO, USA). All channel blockers and PKI_5–24_ were prepared as concentrated stock solutions in distilled water or DMSO and either added immediately to ACSF or the recording pipette at working concentrations or stored at −20 °C for subsequent utilization.

### 2.2. Immunohistochemistry

The immunofluorescence double-labeling procedure followed our previous studies [42,45]. The rats were deeply anesthetized using sodium pentobarbital (50 mg/kg, i.p.) and transcardially perfused with 37 °C saline followed by 4% ice-cold paraformaldehyde (PFA) in 0.01M PBS. Brains were post-fixed with 4% PFA for 2 h at 4 °C and then dehydrated with 30% *wt*/*vol* sucrose in 0.01 M PBS for 72 h. Coronal sections (35 μm) containing the medial prefrontal cortex were cut with a Leica CM900 freezing microtome. In immunofluorescent staining, sections were incubated for 30 min with 0.5% Triton X-100 and then blocked in 10% Normal Goat Serum (Invitrogen, Carlsbad, CA, USA) in 0.01M PBS for 1 h at room temperature. Sections were incubated overnight at 4 °C with primary antibody against rabbit anti-α2A-AR (Novus Biological, Centennial, CO, USA. Cat. # R-1034-1000, 1:800), mouse anti-NeuN (Millipore, Billerica, MA, USA. Cat. # MAB377B, 1:500), mouse anti-CaMKII (Chemicon International, Temecula, CA, USA. Cat. # MAB8669, 1:120), mouse anti-MAP2 (Millipore, Cat. # MAB364, 1:600), or mouse anti-vGLUT1 (Millipore, Cat. # MAB5502, 1:500) in the blocking buffer. Selecting appropriate Alexa dye-conjugated secondary antibodies ensured no crosstalk between fluorescent dyes or cross-reactivity between secondary antibodies (Jackson Immunoresearch, West Grove, PA, USA). Sections were incubated with secondary antibody at room temperature for 2 h. After being washed with 0.01 M PBS three times, sections were mounted using gelatinized glass slides and then coverslipped with FluoromountTM (Catalog No. F4680, Sigma-Aldrich, St. Louis, MO, USA). Images were acquired using an Olympus FV 1000 confocal laser-scanning microscope and analyzed using Image J (ImageJ 1.x), as previously described [42].

### 2.3. Whole-Cell Patch-Clamp Recordings

The rats were deeply anesthetized with sodium pentobarbital (50 mg/kg, i.p.). After decapitation, the brain was rapidly removed and submerged in artificial cerebrospinal fluid (ACSF) containing (in mM) 119 NaCl, 2.5 KCl, 1 CaCl_2_, 3 MgSO_4_, 1 NaH_2_PO_4_, 26.2 NaHCO_3_, and 11 glucose and bubbled with 95% O_2_–5% CO_2_. The brain was cut into coronal slices (300 μm in thickness) containing medial prefrontal cortex (mPFC) using a manual Vibratome Sectioning System (1000+, Pelco 102, Ted Pella, Redding, CA, USA). The brain slices were collected and incubated immediately with an oxygenated warm ACSF and allowed to recover for 30 min at 32 °C. Then, slices were held at room temperature for at least 1 h before recording.

The mEPSC/mIPSC recording and analysis were conducted according to our previous studies [38,45,46]. Whole-cell recordings were performed in layers 5–6 of the pyramidal neurons of the mPFC. Patch pipettes (3–5 MΩ) were pulled from the borosilicate tubing (#B150-86-10; Sutter Instruments, Novato, CA, USA) by the horizontal micropipette puller (P-97, Sutter Instruments). For mEPSC recordings, the internal pipette solution contained (in mM) 150 K-gluconate, 0.4 EGTA, 8 NaCl, 2 ATP-Mg, 0.1 GTP-Na_3_, 10 HEPES, and 10 Na_2_-phosphoreatine. For mIPSC recordings, the internal pipette solution contained (in mM) 75 K-gluconate, 75 KCl, 0.4 EGTA, 8 NaCl, 2 ATP-Mg, 0.1 GTP-Na_3_, 10 HEPES, and 10 Na_2_-phosphoreatine with pH adjusted to 7.2–7.4 by KOH, and it had an osmolarity of 290–320 mOsm.

Whole-cell patch-clamp under voltage-clamp mode was performed to record postsynaptic currents with an Axon 200B amplifier (Molecular Devices, Sunnyvale, CA, USA). Recordings were digitized at 10 kHz and filtered at 2 kHz. The series resistance (Rs) was monitored continuously by applying a voltage pulse throughout the experiment. Data were discarded if the Rs of a recorded cell changed by 20%. For the recording of miniature excitatory postsynaptic currents (mEPSCs) and miniature inhibitory postsynaptic currents (mIPSCs), TTX (1 μM) was applied to block voltage-activated sodium channels. Also, mEPSC was recorded in the presence of picrotoxin (100 μM) to block GABAergic transmission in the recorded neurons. mIPSCs were recorded in the bath of DNQX (20 μM) and AP5 (50 μM) to block glutamatergic transmission in a recorded neuron. To inhibit the α2A-ARs in the recorded neurons, we loaded the membrane-impermeable PKA inhibitor, protein kinase A inhibitor fragment 5–24 (PKI_5–24_, 0.5 μM), into the recording pipette during mEPSC and mIPSC recordings. Both of them were recorded at holding potentials of –70 mV. Data were analyzed using Mini Analysis software (v6.0). The effect of the agonist was assessed 5 min after the start of the application.

### 2.4. Depletion of Catecholamine

Our previous work described the method in detail [38]. Reserpine, an inhibitor of the vesicular monoamine transporter, was applied to deplete presynaptic catecholamine vesicles. Rats were pre-treated with reserpine (1.5 mg/kg, i.p.) 2 h before the brain slice preparation. Additionally, brain slices were incubated in ACSF containing reserpine (10 μM) for at least 1 h and electrophysiologically recorded in the same solution.

### 2.5. Confocal Microscopy

Immunolabeled brain sections were imaged using a Leica SP2 confocal laser-scanning microscope (Leica Microsystems, Mannheim, Germany). Fluorophores were excited at 488 nm and 543 nm with emission signals collected through bandpass filters of 505–530 nm and 560–615 nm, respectively. Twelve-bit images were acquired at a resolution of 1024 × 1024 pixels using either a 20× objective or a Plan-Apochromat 63×/1.4 oil-immersion objective. Immunoreactivity was assessed under optimal imaging conditions, including a reduced pinhole size (set to 1.5 Airy units), thin optical slices, and a high numerical aperture lens to minimize background cytoplasmic fluorescence. To ensure accurate comparison of protein distribution (e.g., α2A-AR, NeuN, CaMKII, MAP2, vGluT1, and GAD65), all confocal parameters, including laser intensity, pinhole diameter, and photomultiplier gain, were kept constant across samples. Image processing was limited to uniform linear adjustments in brightness and contrast using ImageJ (ImageJ 1.53t). No immunosignal was observed in control sections where the primary antibody was omitted. A multi-tracking acquisition mode was employed to avoid spectral bleed-through and ensure clear separation of fluorescence channels. Representative images were selected based on reproducibility across multiple sections and animals, ensuring that the presented figures faithfully reflect the overall staining patterns observed in the experiments.

### 2.6. Data Analysis and Statistics

The frequency and amplitude of mEPSC/mIPSC were analyzed using the Mini Analysis software package (v6.0, Synaptosoft, Leonia, NJ, USA). Events above a fivefold baseline noise level in amplitude and 10–90% rise time of less than 2 ms were detected and used for analysis. In our recordings, mEPSC events with an amplitude greater than 5-fold baseline were detected and used for statistical analysis. For each cell, guanfacine-induced changes in cumulative fractions of mEPSC/mIPSC amplitude and inter-event intervals were analyzed for statistical significance using the Kolmogorov–Smirnov (K-S) test (Mini Analysis v8.0) with a conservative critical probability level of *p* < 0.01. All grouped data were analyzed using paired *t*-tests and a critical probability of *p* < 0.05 (Sigmaplot 10.0). Data are expressed as the means ± standard error of the mean (SEM) in the text and figures.

## 3. Results

The results demonstrated that α2A-ARs are expressed at the axonal terminals of glutamate neurons and GABA neurons in the PFC, and pharmacological stimulation of α2A-ARs, located at the presynaptic membrane, reduced the frequency of mEPSCs and mIPSCs, even after the depletion of presynaptic NE vesicles, suggesting a reduction in glutamate and GABA release on pyramidal neurons in layers 5–6 of the PFC.

### 3.1. The α2A-ARs Are Expressed in the Pyramidal Neurons of PFC

To identify the expression of α2A-ARs in pyramidal neurons of PFC, we examined the expression of α2A-ARs using the well-established three neuron-specific markers: (1) NeuN, which is immunoreactive for almost all types of neurons [45]; (2) CaMKII, a marker of glutamatergic pyramidal neuron cerebral cortex [47,48]; and (3) MAP2, a type of microtubule-associated protein, which is a neuron marker specifically for labeling neurites [46,49,50,51]. The specificity of the α2A-AR antibody used in the present study was identified in our lab [42].

**Figure 1:**
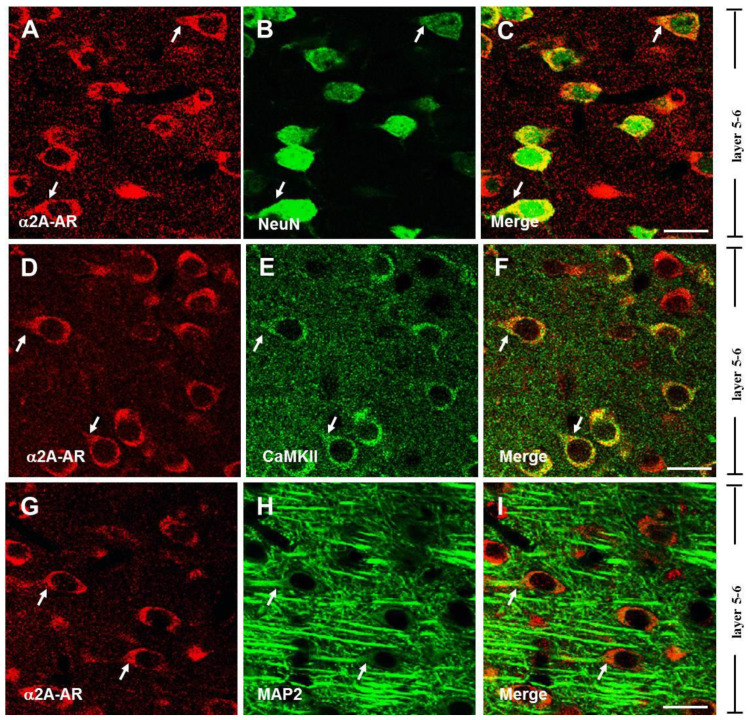
Expression of α2A-ARs in pyramidal neurons in layers 5–6 of the PFC. Single-plane confocal images show the α2A-AR-ir (**A**,**D**,**G**), NeuN-ir (**B**), CaMKII-ir (**E**), and MAP2-ir (**H**). Merging of the paired images (**A**,**B**), (**D**,**E**), and (**G**,**H**) shows the double-labeled neuron. Double-labeled elements of red (α2A-AR) and green (NeuN, CaMKII, and MAP2) appear in yellow (**C**,**F**,**I**). Arrowheads indicate a double-labeled neuron. Scale bar, 20 μm.

As shown in Figure 1, single-plane confocal images showed that α2A-AR immunoreactive (α2A-AR-ir) appeared on the membrane (Figure 1C), which was consistent with our previous reports [41]. We double labeled α2A-AR with NeuN, CaMKII, and MAP2, respectively. Merging single-plane images showed that α2A-AR-ir was co-labeled with NeuN-ir (Figure 1A–C), CaMKII-ir (Figure 1D–F), and MAP2-ir (Figure 1G–I), respectively. Thus, in layers 5–6 of the PFC, the majority of α2A-ARs were expressed on neurons expressing NeuN; α2A-ARs were also widely distributed in almost all CaMKII-expressing neurons. In addition, α2A-AR and MAP2 immunoreactivity were observed in overlapping patterns on the soma and proximal neurites, although the signal in neurites was weaker. A similar expression pattern of α2A-ARs was observed in layers 2–3 of the PFC (Supplementary Figure S1). In summary, α2A-ARs are widely distributed in pyramidal neurons and located in their cell bodies and neurites in the PFC.

### 3.2. The α2A-ARs Are Located at the Glutamatergic and GABAergic Terminals in the PFC

**Figure 2:**
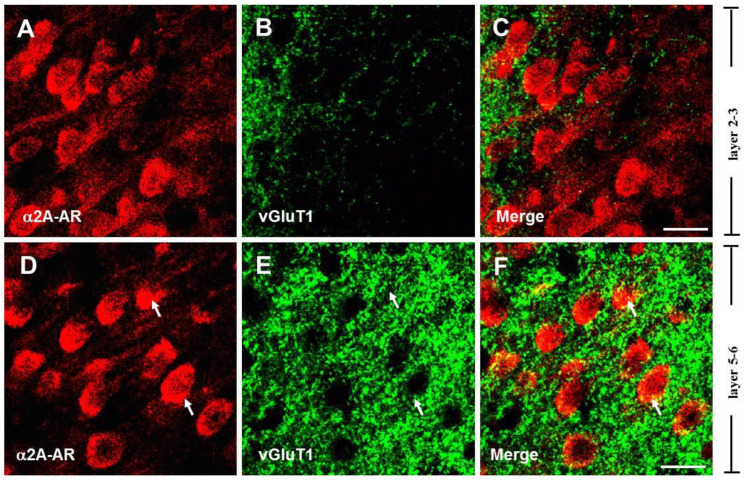
Expression of α2A-ARs on glutamatergic terminals in the PFC. Single-plane confocal images showing the α2A-AR-ir (**A**,**D**) and vGluT1-ir (**B**,**E**). vGluT1-ir appears in punctate structures distributed around the unlabeled soma (**B**,**E**). Merging of the paired images (**A**,**B**) and (**D**,**E**) shows that the puncta of vGluT1 surround the cell bodies of α2A-AR-ir cells in layers 2–3 (**A**) and layers 5–6 (**D**). Partially overlapping areas of red (α2A-AR) and green (vGluT1) profiles appear yellow (**C**,**F**), indicated by arrowheads. Scale bar: 20 μm.

Previous studies have implicated α2A-ARs in the regulation of glutamate release in the cortex [52], suggesting that these receptors may be expressed on the axon terminals of glutamatergic neurons. To identify whether the α2A-ARs are located on glutamatergic terminals in the PFC, we co-labeled the α2A-AR with vesicular glutamate transporter 1 (vGluT1), which is regarded as an essential glutamatergic terminal marker and is required for glutamatergic neurotransmitter transmission [53,54]. Single-plane confocal images showed that vGluT1 immunoreactivity (vGluT1-ir) was present densely in layers 5–6 (Figure 2E). Merging single-plane images showed that α2A-AR-ir and vGluT1-ir were co-located at the border of neurons (Figure 2F). The vGluT1-ir co-labeled with α2A-AR-ir neuron was also found in layers 2–3 (Figure 2B,C). These data demonstrate that the α2A-ARs are expressed in glutamatergic terminals in the PFC.

**Figure 3:**
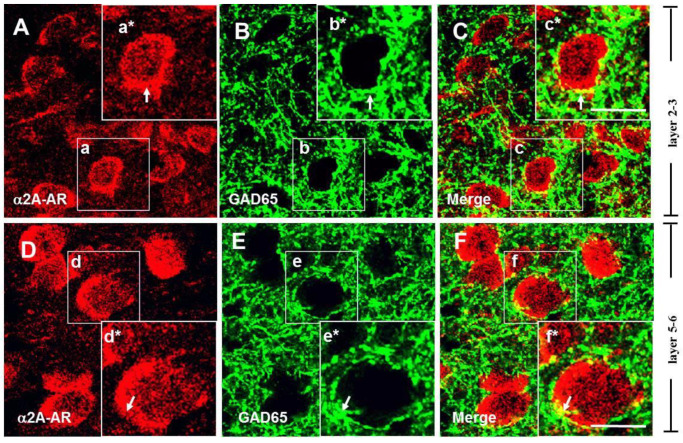
Expression of α2A-ARs on GABAergic terminals in the PFC. Single-plane confocal images show apparent co-localization of α2A-AR (**A**,**D**) with GAD65 (**B**,**E**) in the PFC. Yellow signals (**C**,**F**), indicated by arrows, suggest the potential expression of α2A-ARs in GABAergic terminals. Insets: a*–f* showing enlarged views of white boxes in (**a**–**f**), respectively. Scale bar: 20 μm.

It has been reported that α2A-ARs mediate the presynaptic inhibition of GABAergic transmission induced by NE, as well as the expression of α2A-ARs in GABAergic interneurons of the rat PFC [42], indicating that α2A-ARs may be expressed on the axon terminals of GABAergic neurons [55]. To identify their expression on GABAergic terminals, we co-labeled the α2A-ARs with glutamic acid decarboxylase 65 (GAD65), a GABA synthesis limiting enzyme dominantly located on GABAergic terminals [56]. As shown in Figure 3, single-plane confocal images showed that GAD-ir appeared in punctate structures distributed in the neuropil and around unlabeled cell bodies (Figure 3B,C), consistent with previous reports [45]. The results showed that α2A-AR signals were frequently observed in close apposition or overlapping with GAD65 immunostaining in layers 5–6 of the PFC, suggesting potential expression in GABAergic terminals.

### 3.3. Stimulation of Presynaptic α2A-ARs Suppresses Glutamate/GABA Release in the PFC

**Figure 4:**
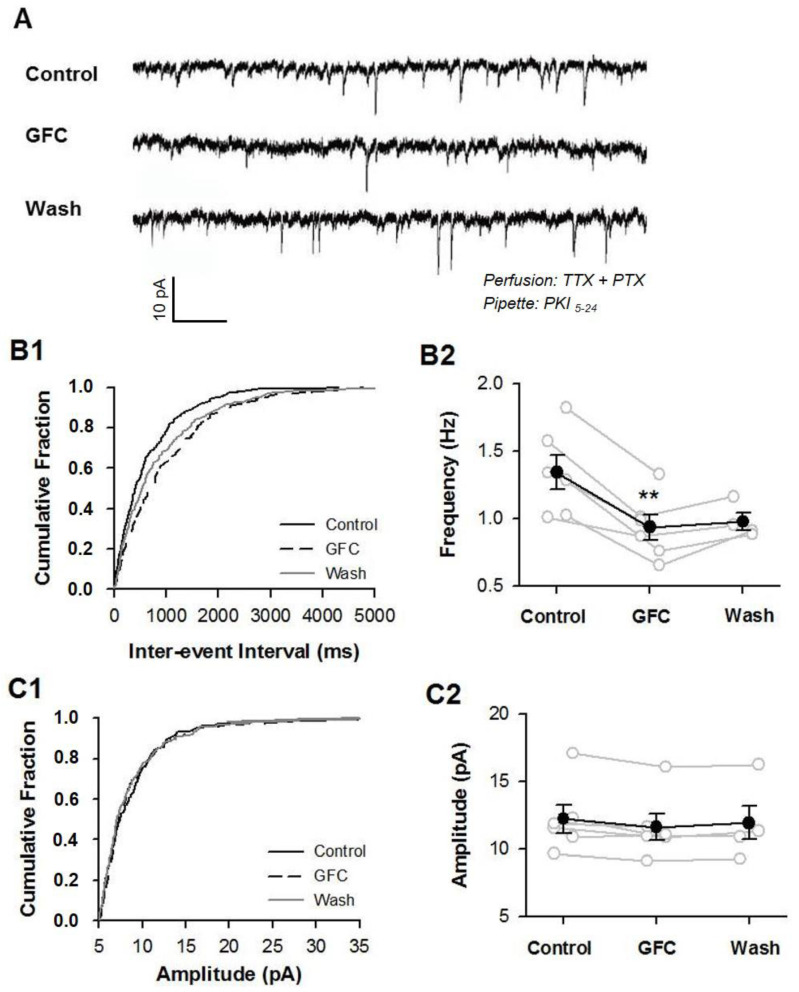
Stimulation of presynaptic α2A-ARs suppresses the release of glutamate in the PFC. Guanfacine suppressed the frequency but did not affect the amplitude of mEPSCs. (**A**) Representative mEPSC traces at −70 mV before, during, and after application of GFC (50 μM). (**B1**) Cumulative probability curves of mEPSCs from a single cell. (**B2**) The individual (open circles) and summary (closed circles) frequency of mEPSCs. (**C1**) Cumulative amplitude curves of mEPSC from a single cell. (**C2**) The individual (open circles) and summary (closed circles) amplitude of mEPSCs. ** *p* < 0.01 for GFC vs. Control, paired *t*-test, n = 6 cells.

**Figure 5:**
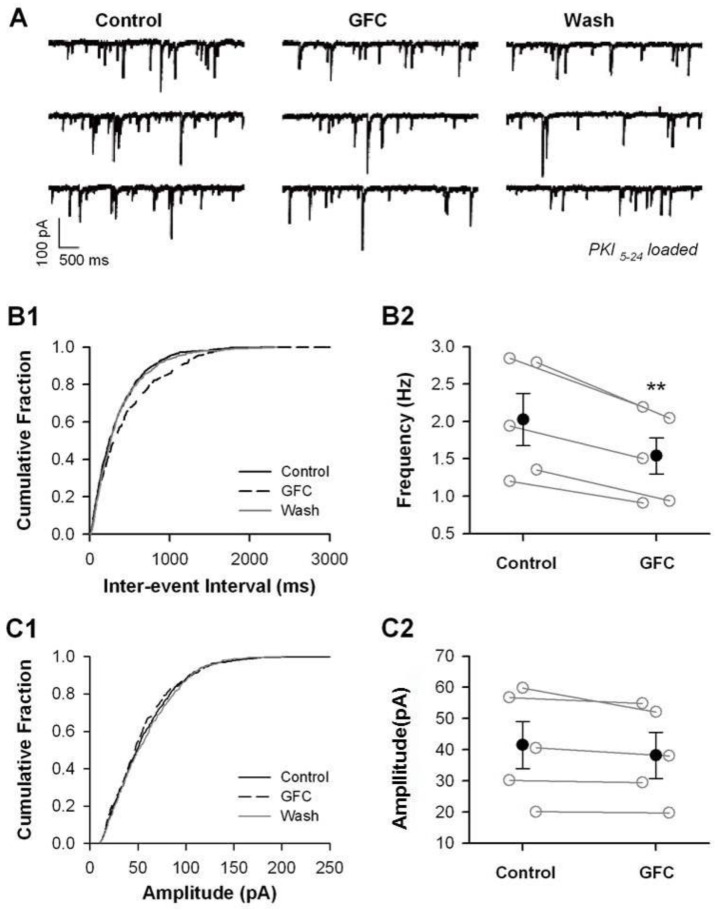
Stimulation of presynaptic α2A-ARs suppresses GABA release in the PFC. Guanfacine suppressed the frequency but did not affect the amplitude of mIPSCs. (**A**) Representative mIPSC traces at −70 mV before and during application of GFC (50 μM). (**B1**) Cumulative probability curves of mIPSCs from a single cell. (**B2**) The individual (open circles) and summary (closed circles) frequency of mIPSCs. (**C1**) Cumulative amplitude curves of mIPSCs from a single cell. (**C2**) The individual (open circles) and summary (closed circles) amplitude of mIPSCs. ** *p* < 0.01 for GFC vs. Control, paired *t*-test, n = 5 cells.

To explore the role of presynaptic α2A-ARs in glutamate and GABA release, we used the α2A-AR selective agonist guanfacine (GFC) in perfusion to activate α2A-ARs. We also incorporated PKI_5–24_ (0.5 μM), a membrane-impermeable inhibitor of PKA, into an internal pipette solution to inhibit postsynaptic α2A-AR function by blocking the PKA signaling pathway [57]. We recorded miniature excitatory postsynaptic currents (mEPSCs) and miniature inhibitory postsynaptic currents (mIPSCs), which correspond to the responses generated by the spontaneous release of single vesicles when action potential firing is blocked using tetrodotoxin (TTX). It is generally understood that the frequency of mEPSCs and mIPSCs reflects presynaptic vesicle release, while their amplitudes indicate the sensitivity of the postsynaptic receptors (Figure 4A and Figure 5A).

Under this experimental condition, we recorded mEPSCs with the presence of picrotoxin (100 μM) to block GABAergic synaptic transmission and TTX (1 μM) to examine the effect of GFC (50 μM) on the frequency and amplitude of mEPSCs (Figure 4A). The results showed that the frequency of mEPSCs was reduced significantly upon GFC application (Figure 4B2; 1.34 ± 0.13 Hz under baseline vs. 0.93 ± 0.10 Hz under GFC; *p* < 0.01, paired *t*-test; n = 6 cells) and the amplitude of mEPSCs was unchanged (Figure 4C2; 12.2 ± 1.04 pA under baseline vs. 11.60 ± 0.96 pA under GFC; *p* > 0.05, paired *t*-test). The GFC suppression showed a reversible tendency after washout of GFC (5/6 cells) (Figure 4B2).

Similarly, mIPSCs were recorded from pyramidal cells of layers 5–6 in the presence of DNQX (20 μM) and AP5 (50 μM) to block glutamatergic synaptic transmission and TTX (1 μM) to examine the effect of GFC (50 μM) on the frequency and amplitude of mIPSCs (Figure 5A). We found that GFC dramatically reduced the frequency of mIPSCs (Figure 5B2; 2.23 ± 0.35 Hz under baseline vs. 1.67 ± 0.28 Hz under GFC; *p* < 0.01, paired *t*-test) and did not affect the amplitude of mIPSCs (Figure 5C2; 36.9 ± 7.89 pA under baseline vs. 35.51 ± 7.45 under GFC; *p* > 0.05, paired *t*-test).

These results indicate that stimulation of presynaptic α2A-ARs suppresses the release of glutamate and GABA.

### 3.4. Suppression of Glutamate/GABA Release by Stimulating Presynaptic α2A-ARs at Non-NE Terminals

Considering the abundant expression of α2A-AR on NE terminals (autoreceptors) in the PFC, we wondered whether the stimulation of presynaptic α2A-AR on NE terminals modulates the glutamate/GABA release in the mPFC. Because it is unable to block presynaptic α2A-ARs on NE terminals specifically, we used an alternative strategy to deplete presynaptic NE vesicles by reserpine, which can block the uptake and storage of NE as well as dopamine into synaptic vesicles by depleting presynaptic catecholamine vesicles irreversibly as a result of inhibiting the vesicular monoamine transporters. Rats were pre-treated with reserpine (1.5 mg/kg, i.p.) before the brain slices were prepared. The brain slices were incubated in ACSF containing reserpine (10 μM) for at least 1 h before recordings. Also, the slices were continuously bathed with ASCF containing reserpine (10 μM) during recordings.

**Figure 6:**
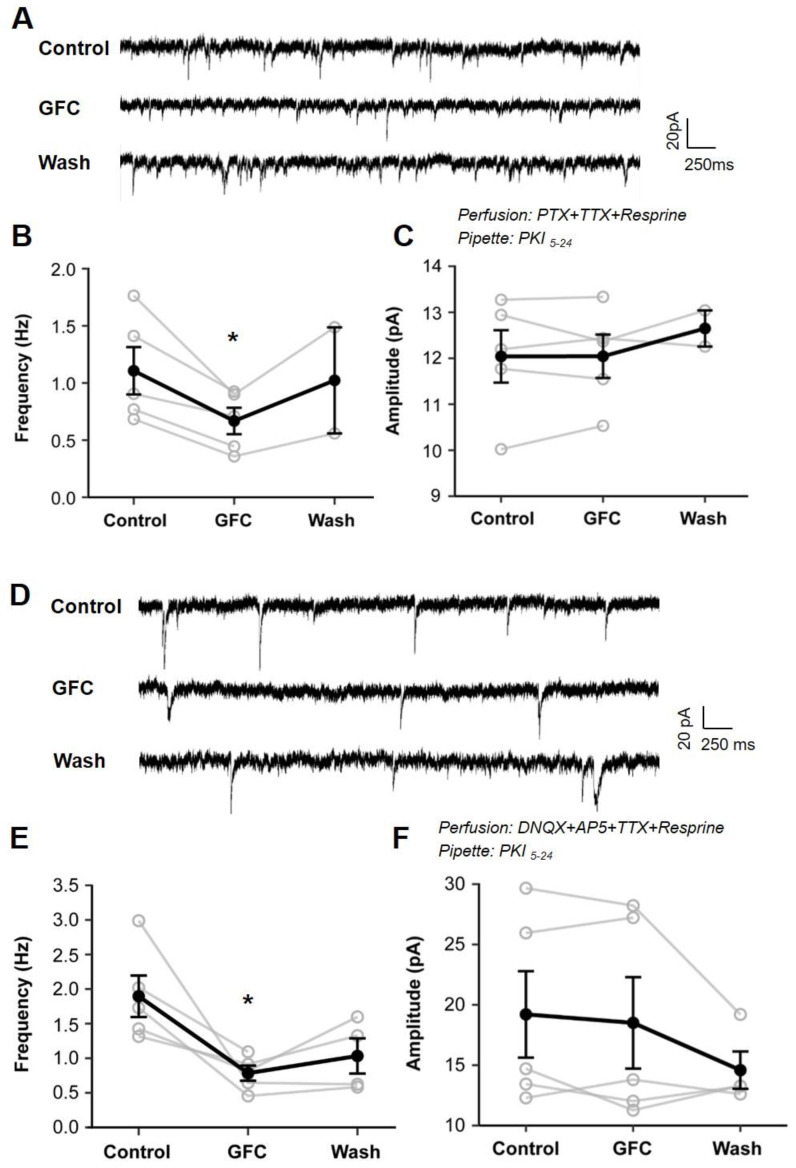
Stimulation of presynaptic α2A-AR at non-NE terminals suppresses glutamate and GABA release in the PFC. Guanfacine suppressed the frequency but did not affect the amplitude of mEPSCs/mIPSCs in NE-depleting slices. (**A**) Representative mEPSC traces at −70 mV before, during, and after application of GFC (50 μM). (**B**,**C**) The individual (open circles) and summary (closed circles) frequency and amplitude of mEPSCs (n = 5). (**D**) Representative mIPSC traces at -70 mV before, during, and after GFC (50 μM) application. (**E**,**F**) The individual (open circles) and summary (closed circles) frequency and amplitude of mIPSCs (n = 5). * *p* < 0.05 for GFC vs. Control, paired *t*-test.

Under this experimental condition and with PKI_5–24_ loaded, we examined the effect of GFC on mEPSCs and mIPSCs (Figure 6A,D). As shown in Figure 6B, GFC significantly reduced the frequency of mEPSC (Figure 6B; 1.11 ± 0.18 Hz under baseline vs. 0.67 ± 0.10 Hz under GFC; *p* < 0.05, paired *t*-test; n = 5) and had no impact on the amplitude of mEPSCs (Figure 6C; 12.04 ± 0.51 pA under baseline vs. 12.65 ± 0.42 under GFC; *p* > 0.05, paired *t*-test). Similarly, the frequency of mIPSCs exhibited a significant decrease upon GFC application (Figure 6E, 1.90 ± 0.27 Hz under baseline vs. 0.78 ± 0.10 under GFC; *p* < 0.05, paired *t*-test; n = 5), and the amplitude of mIPSCs was unchanged (Figure 6F, 19.21 ± 3.2 pA under baseline vs. 18.51 ± 3.41 pA under GFC; *p* > 0.05, paired *t*-test). Thus, although NE vesicles were depleted by reserpine, GFC suppression of mEPSC/mIPSC frequency was similar to results without reserpine treatment.

These results suggest that activating presynaptic α2A-ARs on glutamatergic and GABAergic terminals suppresses glutamate and GABA release, indicating a heterosynaptic role of α2A-ARs in the PFC.

## 4. Discussion

It is widely accepted that presynaptic α2A-ARs are primarily located at NE terminals, where they serve as autoreceptors to regulate NE release in the PFC negatively [23]. However, the current study presents morphological evidence indicating that α2A-ARs are also found at non-NE terminals in the PFC, specifically at axon terminals of excitatory glutamate neurons and inhibitory GABA neurons. Notably, the stimulation of these presynaptic α2A-ARs leads to a decrease in the release of glutamate/GABA onto pyramidal neurons in layers 5–6 of the PFC (Figure 4 and Figure 5), even under conditions in which postsynaptic α2A-ARs are disabled and NE vesicles are depleted (Figure 6). These findings offer new insights into the mechanisms underlying α2A-AR-mediated modulation of cognitive functions in the PFC.

It is well known that α2A-ARs are present in presynaptic NE terminals, which negatively regulate NE release [58,59,60]. The suppression of mEPSC/mIPSC frequency induced by guanfacine was unaffected by the depletion of NE vesicles (Figure 6), which suggests that guanfacine effects do not arise from the inhibition of NE release. In addition, the guanfacine-induced reduction in mEPSC frequency persisted even when the PKA inhibitor PKI_5–24_ was delivered via pipette into the patched postsynaptic cells (Figure 5 and Figure 6). These results indicate that the impact of guanfacine is mediated through presynaptic α2A-ARs on non-NE glutamate and GABA terminals, which are called heteroreceptors. The other brain areas, including the hippocampus, BNST, ventrolateral medulla, and nucleus of the solitary tract (NTS), were consistent with the expression of heterosynaptic α2A-ARs [35,43,44,61]. The α2A-ARs are G protein-coupled receptors (GPCRs) that couple with Gi/o proteins. It is well-established that Gi/o-coupled GPCRs inhibit presynaptic exocytosis by interacting with the βγ subunit of the G-protein (Gβγ). Research suggests that Gβγ mediates the inhibitory effects on exocytosis when presynaptic Gi/o-coupled 5-HT_1B_-like receptors are activated [62,63]. The regulation of glutamate and GABA release by α2A-ARs may follow a comparable process. Evidence has been documented for heterosynaptic modulation of glutamate release by α2A-ARs via direct interaction with Gβγ in the amygdala [64]. Furthermore, the activation of α2A-ARs has been shown to inhibit L-type calcium channels in the retina, thereby reducing calcium influx [65].

The present study demonstrated that guanfacine significantly reduced the frequency of mEPSC/mIPSC without affecting their amplitudes (Figure 3 and Figure 4), indicating that postsynaptic α2A-ARs were completely inhibited by the loading of the membrane-impermeable PKA inhibitor PKI_5–24_ into the recording pipette. Consequently, the decrease in mEPSC frequency induced by guanfacine cannot be attributed to postsynaptic α2A-AR activation. Meanwhile, guanfacine suppression of mEPSC frequency was not observed when NF023, a membrane-permeable Gi protein inhibitor, was introduced into the recording pipette. It is likely that NF023 passed through the membranes and acted on the presynaptic α2A-ARs; that is, the suppression of mEPSC frequency induced by guanfacine (50 μM) mainly arises from activating α2A-ARs but not non-specific effects.

In this study, we found that guanfacine significantly decreased the frequency of mEPSC/mIPSC without altering their amplitudes (Figure 3 and Figure 4), which suggests that the postsynaptic α2A-ARs were fully inhibited by introducing the membrane-impermeable PKA inhibitor PKI_5–24_ into the recording pipette. As a result, the observed reduction in mEPSC frequency caused by guanfacine cannot be linked to postsynaptic α2A-AR activation. Additionally, when NF023, a membrane-permeable Gi protein inhibitor, was introduced into the recording pipette, guanfacine did not reduce mEPSC frequency (Supplementary Figure S2), which indicates that NF023 penetrated the membranes and interacted with presynaptic α2A-ARs, suggesting that guanfacine’s effect on lowering mEPSC frequency (at a concentration of 50 μM) primarily results from the activation of α2A-ARs rather than from non-specific effects.

Previous research has indicated that the stimulation of α2A-ARs inhibits excitatory synaptic transmission in the pyramidal cells of the PFC through a postsynaptic mechanism [46]. The Gi-cAMP–PKA–PP1–CaMKII–AMPAR signaling pathway mediates this inhibition [38]. These studies underscore the inhibitory effects of postsynaptic α2A-AR stimulation on excitatory synaptic transmission. In our present study, we observed a comparable inhibitory effect resulting from the stimulation of presynaptic α2A-ARs on glutamate release (Figure 4) and morphologically identified α2A-ARs on glutamatergic terminals (Figure 2). It could be posited that presynaptic α2A-ARs are implicated in guanfacine suppression of evoked EPSC (eEPSC) by reducing presynaptic glutamate release. However, this hypothesis may not be correct, as eEPSC amplitudes remained largely unaffected following guanfacine treatment, with 93% of the baseline in the PKI_5–24_-loaded condition compared to 67% in the control group [38], indicating that guanfacine’s inhibitory effect on glutamate release contributes negligibly to the suppression of eEPSC amplitude. One possibility is that the stimulation of α2A-ARs primarily influences action potential-independent glutamate release.

Behavioral, pharmacological, and electrophysiological research has established that stimulation of α2A-ARs positively affects cognitive functions in the PFC. Earlier iontophoresis research on actively behaving monkeys demonstrated that activation of α2A-ARs enhances neuronal activity in the PFC related to working memory tasks [12,13]. Specifically, Wang and colleagues revealed that guanfacine enhances delay-related neuronal activity by inhibiting cAMP production, closing HCN channels, and strengthening PFC neural networks [12]. Conversely, research by Ji et al. (2008) using whole-cell and field-potential recordings indicated that the stimulation of α2A-ARs reduces excitatory synaptic transmission in PFC pyramidal cells [46]. Further insights were provided by Yi et al. (2013), who employed whole-cell recordings and Western blotting to elucidate the intracellular mechanisms by which α2A-ARs suppress excitatory synaptic transmission [38]. These studies highlight a decrease in excitatory synaptic transmission due to the activation of α2A-ARs on postsynaptic pyramidal neurons.

Our current study observed a similar inhibitory effect on neurotransmitter release, both excitatory and inhibitory, resulting from activating α2A-AR at presynaptic non-NE terminals. These results suggest that the guanfacine-driven enhancement of PFC neuronal firing during the delay period in working memory tasks and the suppression of excitatory/inhibitory synaptic transmission may involve distinct mechanisms. These mechanisms are likely determined by the specific distribution of α2A-ARs in the PFC. For example, postsynaptic α2A-ARs located near HCN channels could be responsible for modulating neuronal firing during working memory tasks. Furthermore, postsynaptic α2A-ARs in neurons specific to certain inputs may restrict excitatory inputs via the Gi-cAMP–PKA–CaMKII–AMPAR signaling pathway. Through this mechanism, α2A-AR protects PFC functions from the insult of stress, such as PFC neurons receiving projections from the related brain regions. On the other hand, presynaptic α2A-ARs located on non-NE glutamate and GABA terminals might form synaptic connections with dendritic spines, allowing for precise regulation of neuronal network functions in the PFC. In support of this, the α2A-AR agonist has been reported to increase the possibility of dendritic Ca^2+^ events in the tuft region and lower the threshold for dendritic Ca^2+^ spikes via decreasing I_h,_ thereby strengthening the functional connectivity of PFC networks [25,26].

Our research provides important insights into the presynaptic and heteroreceptor roles of α2A-ARs in the PFC, though it has some limitations. Firstly, relying solely on vGluT1 as a marker might not fully represent the diversity of glutamatergic subtypes, since vGluT1 and vGluT2 demonstrate complementary expression patterns in the brain [53,54]. Some neurons in the PFC may express both markers, highlighting diversity within glutamatergic populations. Future studies should incorporate vGluT2 labeling or dual vGluT1/vGluT2 approaches to elucidate the roles of α2A-ARs among different excitatory neurons.

Secondly, the absence of in vivo behavioral validation complicates the connection between our electrophysiological findings and cognitive outcomes. Future research should incorporate behavioral assays to evaluate the impact of presynaptic α2A-AR activation on cognitive functions such as working memory and attention. Employing siRNA technology or genetically modified animal models could help clarify the relationship between these receptors and cognitive processes. Moreover, combining siRNA with electrophysiological recordings and two-photon imaging could improve our understanding of the role of α2A-ARs in PFC connectivity, which is vital for working memory.

Finally, while our study concentrated on the PFC, α2A-ARs are also found in other brain regions. Future investigations should examine similar mechanisms in regions like the hippocampus, amygdala, and cerebellum. Exploring the downstream signaling pathways of α2A-ARs in various presynaptic terminals could provide insights into how these receptors influence neurotransmitter release and overall brain function.

## 5. Conclusions

Our data provide a morphological and electrophysiological basis for a potential mechanism by which several α2A-AR-mediated improvements in brain cognitive functions occur via heterosynaptic α2A-ARs on non-NE terminals.

## Data Availability

Data presented in the article are available upon reasonable request.

## Supplementary Materials

The following supporting information can be downloaded at: https://www.mdpi.com/article/10.3390/biomedicines13061322/s1, Figure S1: Expression of α2A-ARs in pyramidal neurons in layers 5–6 of the PFC; Figure S2: Effect of α2A-AR stimulation on the frequency and amplitude of mEPSCs with recording pipette loading NF023, a membrane-permeable Gi inhibitor.
