# Supplementary material for: Heterosynaptic Regulation of α2A-Adrenoceptors on Glutamate/GABA Release in the Prefrontal Cortex of Rats
Source: Biomedicines. 2025 May 28;13(6):1322. doi: 10.3390/biomedicines13061322 (PMC12189517; doi:10.3390/biomedicines13061322)
Supplement: Supplementary file 1 [file biomedicines-13-01322-s001.zip › Supp. figure legends.pdf]

Figure S1. Expression of α2A-ARs in pyramidal neurons in layers 2-3 of PFC.

Single-plane confocal images showing the α2A-AR-ir (A, D, G), NeuN-ir (B), CaMKII-ir (E), and MAP2-ir (H). Merging of the paired images (A and B), (C and D), and (G and H) shows the double-labeled neuron. Double-labeled elements of red (α2A-AR) and green (NeuN, CaMKII, and MAP2) appear yellow. Arrowheads indicate a double-labeled neuron. Scale bar, 20 μm.

Figure S2. Guanfacine does not affect the frequency and amplitude of mIPSC when the recording pipette is loaded with NF023, a membrane-permeable Gi protein inhibitor.

(A) Representative mEPSC traces at -70 mV before, during, and after application of GFC (50 μM) with loading NF023 (10 μM) in the recording pipette. (B1, C1) Cumulative probability curves of mEPSC frequency and amplitude from a single cell. (B2, C2) The individual (open circles) and summary (closed circles) frequency and amplitude of mEPSCs (n=5). (D) Representative mIPSC traces at -70 mV before, during, and after GFC application. (E-F) The individual (open circles) and summary (closed circles) frequency and amplitude of mIPSCs (n=5). $p > 0.05$ for GFC *vs*. Baseline, paired *t*-test.
