# Supplementary figures and images for: Heterosynaptic Regulation of α2A-Adrenoceptors on Glutamate/GABA Release in the Prefrontal Cortex of Rats
Source: Biomedicines. 2025 May 28;13(6):1322. doi: 10.3390/biomedicines13061322 (PMC12189517; doi:10.3390/biomedicines13061322)

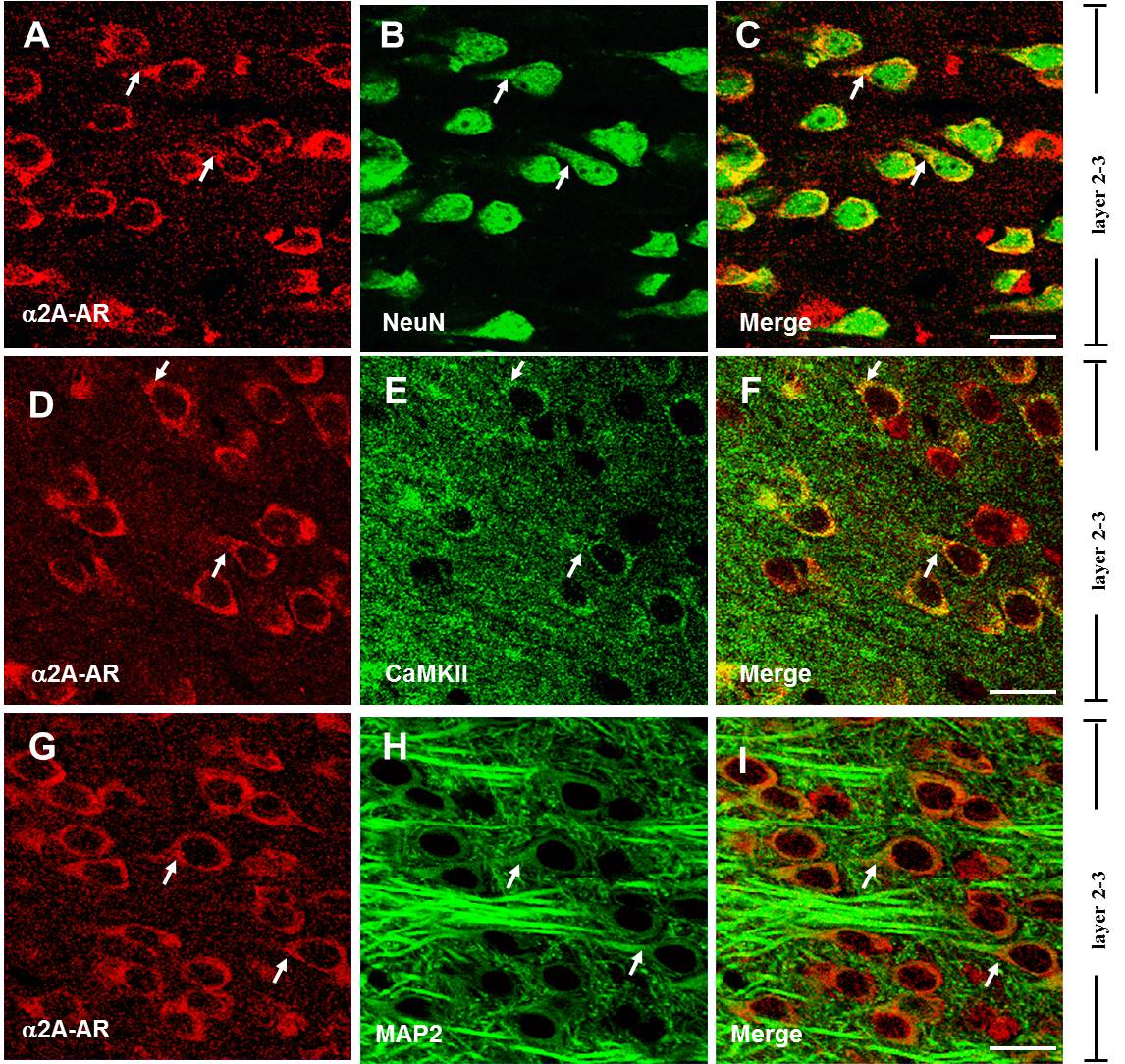

Supplement: Supplementary file 1 [file biomedicines-13-01322-s001.zip › Suppl. figure 1.jpg]

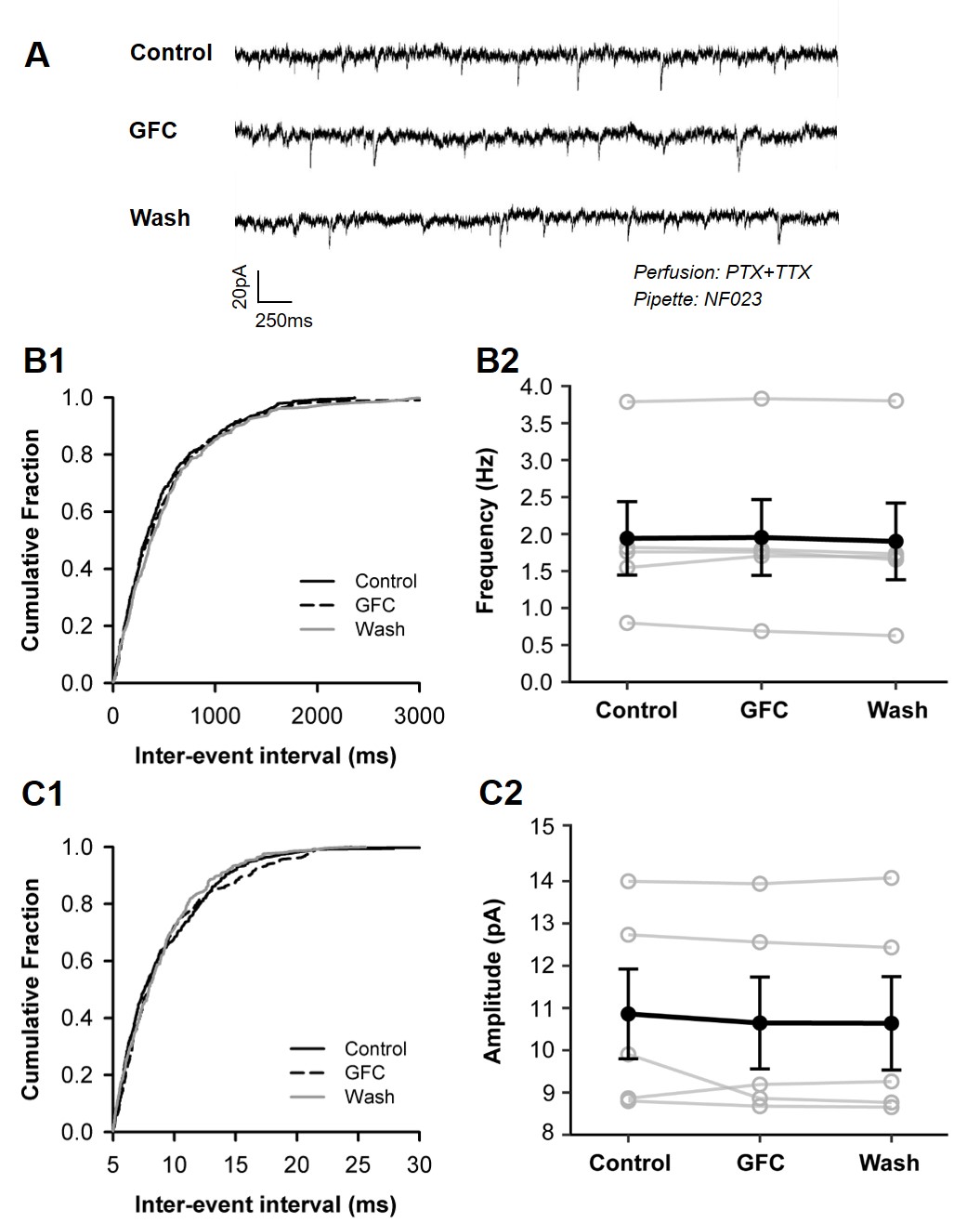

Supplement: Supplementary file 1 [file biomedicines-13-01322-s001.zip › Suppl. Figure 2.jpg]
